# Factors Associated with Early Intervention Intensity for Children Who Are Deaf or Hard of Hearing

**DOI:** 10.3390/children9020224

**Published:** 2022-02-08

**Authors:** Jareen Meinzen-Derr, Meredith E. Tabangin, Mekibib Altaye, Jennifer Ehrhardt, Susan Wiley

**Affiliations:** 1 Division of Biostatistics and Epidemiology, Cincinnati Children’s Hospital Medical Center, Department of Pediatrics, University of Cincinnati College of Medicine, Cincinnati, OH 45229, USA; meredith.tabangin@cchmc.org (M.E.T.); mekibib.altaye@cchmc.org (M.A.); 2 Division of Developmental and Behavioral Pediatrics, Cincinnati Children’s Hospital Medical Center, Department of Pediatrics, University of Cincinnati College of Medicine, Cincinnati, OH 45229, USA; jennifer.ehrhardt@cchmc.org (J.E.); susan.wiley@cchmc.org (S.W.)

**Keywords:** early intervention enrollment, pediatric hearing loss, intervention intensity

## Abstract

We quantified the intensity of early intervention (EI) services allocated to 1262 children who were deaf or hard of hearing (DHH) within a state program and identified factors associated with intervention intensity. Child specific data were collected on children born between 2008 and 2014. Data from Individualized Family Service Plans of children enrolled in Part C EI programming were evaluated for the type and duration of services during their EI enrollment. Associations between EI intensity and child/family variables were examined. Median age of EI enrollment was 5.3 months. The most frequently received services included primary service coordination, specialized DHH service, special instruction, language therapy, and family training; 60% of children received 4 or more different EI services. The median service intensity was 138.1 min per month across all EI years. The factors associated with higher EI intensity included severe hearing loss, bilateral hearing loss and presence of a disability. Children enrolled in EI at later ages received higher intensity of specialized DHH services, suggesting a need to “catch up” due to late acquisition of services. Evaluating EI service intensity broadens our understanding of effective components of state-based programs that support the developmental needs of children who are DHH.

## 1. Introduction

Early intervention (EI) refers to the process of providing services, education and support to infants and toddlers who have disabilities, developmental delays, or are at high-risk for delays. The Part C EI program of the Individuals with Disabilities Education Act [1] is a large EI program which serves infants and toddlers from birth to 3 years once they have been identified with a medical condition, developmental delay, or a condition that places them at high risk for developmental delays (in some states) [2]. EI includes a wide range of services (i.e., home visits, family training, counseling, special instruction, and therapy) for approximately 3% of infants and young children nationally who have disabilities or developmental delays [3]. The intensity of EI programming is a critical factor in ensuring significant and long-term benefits [4]. 

Approximately 6000 infants are born annually in the United States with a permanent hearing loss that without early identification and appropriate intervention, could impact language and communication [5,6]. Every state has established an Early Hearing Detection and Intervention (EHDI) program to help identify these infants who are deaf or hard of hearing (DHH) as early as possible. The American Academy of Pediatrics’ (AAP) EHDI has also endorsed national guidelines that a child should receive: a hearing screening no later than one month of age; a diagnosis no later than 3 months of age; and entry into EI services no later than 6 months of age. Though it varies by state and by year, overall approximately 60% of infants identified with a hearing loss are enrolled into EI [5]. The impact of early identification and subsequent intervention has been noted with improvements in language development in children who are DHH, which can be linked to subsequent academic and social-emotional wellbeing.

The level and intensity of EI services are critical components and depend on a variety of factors. For children who are DHH, additional attention to development that may be impacted by reduced hearing is warranted. Although the service varies from state to state, EI for children who are DHH can focus on communication, audiologic needs (receiving hearing devices, such as hearing aids) and linking to resources that are available to families who have a child who is DHH. The Joint Committee on Infant Hearing, which includes AAP as a member, published a guidance on early intervention for children who are DHH, which highlighted critical factors, such as specialized hearing services and family inclusion, in goal-setting and services for this population of children [7]. There is also robust evidence focused on language supporting the importance of the age of enrollment into EI [8,9,10,11]. Few studies have assessed whether aspects of EI, including dosage or intensity, are associated with outcomes in children who are DHH [12,13,14]. 

In Part C programming, children with greater needs based on developmental profiles will receive more services (more types and more frequency) over a longer duration of time. Children in Ohio who are DHH are automatically eligible for services due to their hearing loss, not because a delay is identified. In fact, a goal of EI for DHH infants is to prevent delays (specifically language and communication) from occurring. Therefore, we were interested in understanding the EI service model that specifically addressed this proactive and habilitative or preventive approach to service delivery. The objectives of this study were to (a) characterize EI services types and intensity for DHH children born in the state of Ohio; (b) evaluate changes over time in service intensity; (c) identify child and family characteristics associated with service intensity. Because DHH infants and toddlers were eligible to specialized services in the state of Ohio, we also specifically reported the intensity of these services. 

## 2. Materials and Methods

### 2.1. Data Sources

Detailed information on the Ohio Data Linkage Project, describing the linkage methodology and data for the present study, has been published previously [15,16] and is summarized briefly here. The Ohio Data Linkage Project was a multi-agency collaboration that successfully linked data from two state-level public health databases to better understand outcomes in children who are DHH born between 2008 and 2014. Child and family characteristics were collected within the birth record and the hearing screening record. The specific characteristics included the child’s race/ethnicity, gestational age, birth weight, and the child’s sex. Race/ethnicity was collected from the birth certificate. Because one of the categories provided to us was “other”, we collapsed “other” and “unknown” categories into “unknown”. The final race categories included white, black/African American, Asian/other, and unknown. Among the “Asian/other” category, three infants were identified as Native American, two as Filipino, and one as Native Hawaiian. The remaining included Asian American, Asian other, Japanese, Korean, and Vietnamese. The family characteristics included maternal and paternal education level and insurance status of infant at time of birth. The data collected as part of the hearing screening database included severity and laterality of hearing loss, age of hearing loss confirmation, and presence of risk indicator(s) for hearing loss. The factors collected as part of EI included dates of the Individualized Family Service Plan (IFSP), presence of a disability diagnosed by a medical professional, and identified developmental delays. The age at EI entry was categorized as less than 6 months, 6 to <12 months, 12 to <24 months, and 24 to 36 months. Between 2008 and 2014, 1746 infants were identified in the state of Ohio with a permanent hearing loss; 1262 (72%) were enrolled into EI.

### 2.2. EI Services

At the time of this study, children in Ohio who were DHH were eligible to receive specialized EI services that followed the SKI-HI Curriculum [17], which uses family-centered programming for infants and young children who are DHH. Services were provided through a regionalized program, with 9 separate regions across the state supporting 88 counties. EI services were identified within a child’s IFSP and were provided primarily in the home. The components of the service type that made up specialized DHH services are illustrated in Table A1. In addition to the DHH Service type, children also received service types based on individual needs. Such services could include (but are not limited to) occupational therapy, physical therapy, speech-language therapy, audiological services, and service coordination. Because of the numerous different types of services a child could receive, we determine a priori to focus on a set of services found most frequently within the IFSP of children who were DHH. These EI services included service coordination, specialized DHH services, special instruction, speech–language, family training and counseling, physical therapy, occupational therapy, audiologic services, and parent education. These services also had the highest cumulative hours of service assigned for our target population of children who were DHH.

Within the IFSP, each service had the following variables we considered as part of the service intensity definition: the interval (e.g., weekly, monthly), frequency (e.g., once, twice), duration of encounter (e.g., 15, 30, 60 min), and duration of service (start and end dates of the service within the IFSP). The time was defined as the IFSP start date to the end date of the service or the end of EI. The overall service exposure was described in the number of different select services, total number of EI hours, and total hours of exposure by service type. The service intensity (outcome) was calculated as the total minutes for each service type divided by total duration of services in months. The EI service intensity, overall and for each selected EI discipline, was determined by summing the total minutes of all services received and dividing by the number of months the child was enrolled. Service-specific minutes were calculated using the start and end dates for that service listed within the IFSP. Service-specific minutes were summed across all services received as the numerator. The denominator was defined by the EI enrollment and exit dates.

### 2.3. Statistical Analysis

Descriptive statistics were calculated on all variables and used to summarize the characteristics of children who were DHH and served by EI. Distributions were assessed for normality. Due to the skewed data distribution, we calculated medians with interquartile ranges (IQR) to describe EI service intensity. The overall EI service use included all common EI services described earlier. 

We evaluated intensity (the outcome) in three ways: (1) at the first assigned IFSP; (2) for the first year a child was enrolled; and (3) for the whole duration a child was enrolled. We evaluated intensity for all identified common services as well as the specialized DHH services. 

Associations between EI intensity and child and family variables were evaluated using the Wilcoxon Rank Sum test. Because the EI intensity variables were found to be skewed, we conducted a log transformation on these variables prior to conducting any regression analyses. We modeled the relationship between demographic and clinical characteristics and the intensity of services received during early intervention using general linear models (GLM) and accounting for clustering in nine regions through generalized estimating equations (GEE) with an exchangeable covariance structure. Models were developed for EI intensity within the first EI year and across all years combined for all common services as well as for the DHH service type. Characteristics associated with service intensity in bivariate analyses with *p* < 0.20 were entered into the GLM models. Stepwise backward elimination was used for variable selection; variables were tested in the model at *p* < 0.10 and stayed in the model at *p* < 0.05. Characteristics associated with intensity in either the first year or the entire early intervention period model were retained in both models. We treated region as both a random and fixed effect to account for potential data correlation within the region and to estimate the contribution of region on the intensity, respectively. Final models for all common services included age of EI entry, race, presence of a hearing loss severity, laterality, disability diagnosis, presence of an identified delay, and region of EI service. Models for specialized DHH Services included for age of EI entry, race, hearing loss severity (severe or profound vs. mild or moderate), laterality (bilateral vs. unilateral), and region of EI service. Least square means with corresponding 95% confidence intervals were reported for EI intensity on the original scale (minutes per month). The SAS version 9.4 (SAS Institute, Cary, NC, USA) was used to conduct all analyses.

## 3. Results

### 3.1. Participants

Of the 1262 children enrolled in EI, the majority of children were white and fewer than half had private insurance (vs. Medicaid) at birth. The median age of enrollment into the EI program was 5.3 months (IQR 3.2–9.3) with over half enrolled before 6 months of age. Although children in this sample were eligible for EI services according to the diagnosis of hearing loss, over 26 also had an additional diagnosed disability. While in EI, one third had a skill delay in one of 5 domains identified while they were in EI; nearly 10% of children had communication as the only delay identified. Demographic characteristics are presented in Table 1 and EI information in Table 2. 

### 3.2. Services Received

**Figure 1:**
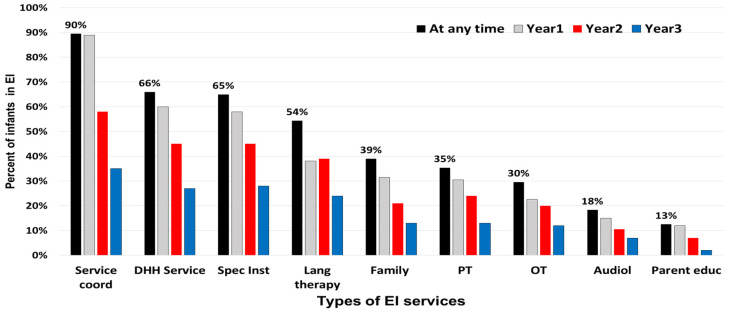
Percent of DHH infants and children assigned to types of early intervention services overall and by early intervention year. Services include: Service coord = coordination, Spec Inst = special instruction, DHH Service = specialized DHH services, Spec Inst = specialized instruction, Lang therapy = speech-language therapy services, Family = family training and counseling, PT = physical therapy, OT = occupational therapy, Audiol = audiology, and Parent educ = parent education.

**Figure 2:**
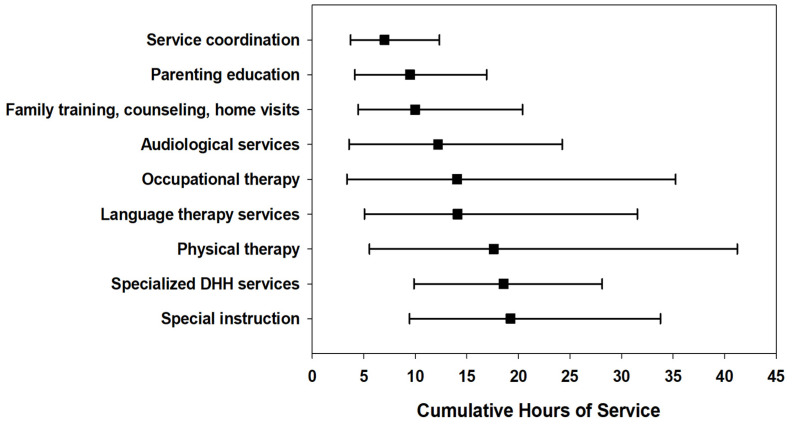
Median cumulative hours of early intervention service for the duration of early intervention, by common service types among children who are deaf or hard of hearing. Error bars represent the 25th and 75th percentiles.

The majority of children (*n* = 760, 60.2%) received four or more EI services. Primary service coordination, specialized DHH service, special instruction, speech–language therapy, and family training were the most frequently received services (Figure 1). In the first year of EI, 89% (*n* = 1128) of children received service coordination while 60% (*n* = 756) of children received specialized DHH services. A little over half of children (54.4%) received speech–language services at least once while in EI; 37.9% (*n* = 478) received services in the first year. Half of the cohort (*n* = 627) remained in EI to year 3. The overall median cumulative hours per child for common services combined was 56.8 [28.4–111.7] with 25% of families receiving 112 h or more throughout EI duration. The median cumulative hours for the specialized DHH service type was <20 h (66% of children received this service at least once). Figure 2 illustrates the median cumulative hours for the duration of EI by service type.

### 3.3. Service Intensity–All Selected Services

**Figure 3:**
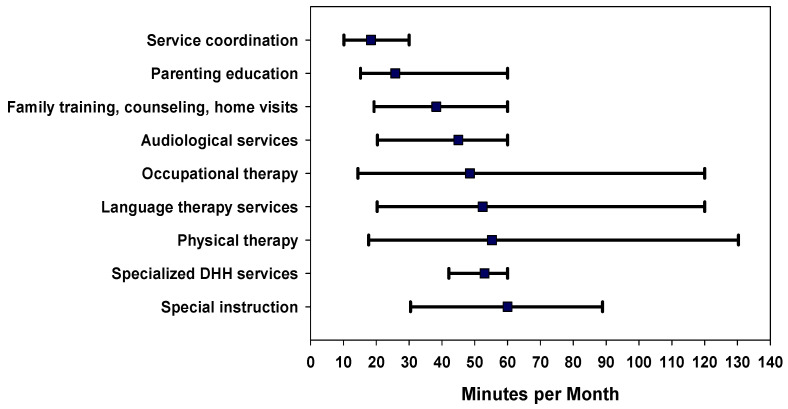
Median intensity as minutes per month of early intervention services per child by service type, for the duration a child was enrolled in early intervention. Error bars represent the 25th and 75th percentiles.

The median (interquartile range [IQR]) for service intensity at the first IFSP (minutes per month) for all services combined was 73.3 [48.0–126.7]. Intensity increased to 121.2 [75.6–204.6] minutes per month in the first year of EI and was 138.1 [82.3–262.1] minutes per month across all years of EI service. Figure 3 illustrates the EI intensity by each common service. The factors significantly associated with overall higher EI intensity (more minutes per month) in unadjusted analyses included prematurity, risk indicator for hearing loss at birth, severe or profound degrees of hearing loss, bilateral hearing loss, and presence of disability (Table 3).

**Figure 4:**
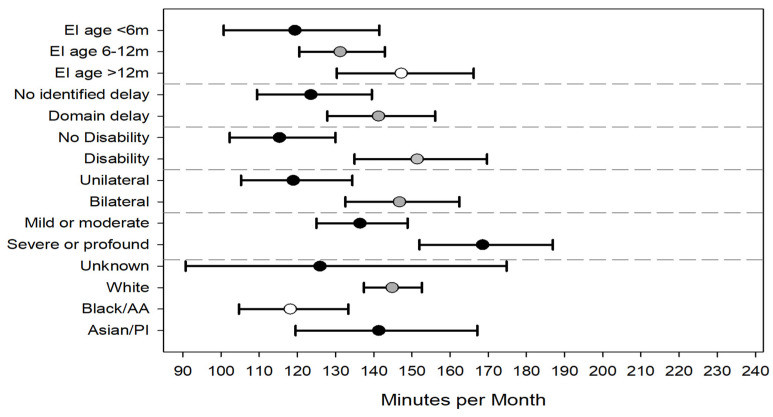
Adjusted average intensity (minutes per month) of all common early intervention service types for first enrollment year. Error bars represent 95% confidence intervals. Abbreviations: EI = early intervention, m = months, AA = African American, and PI = Pacific Islander.

**Figure 5:**
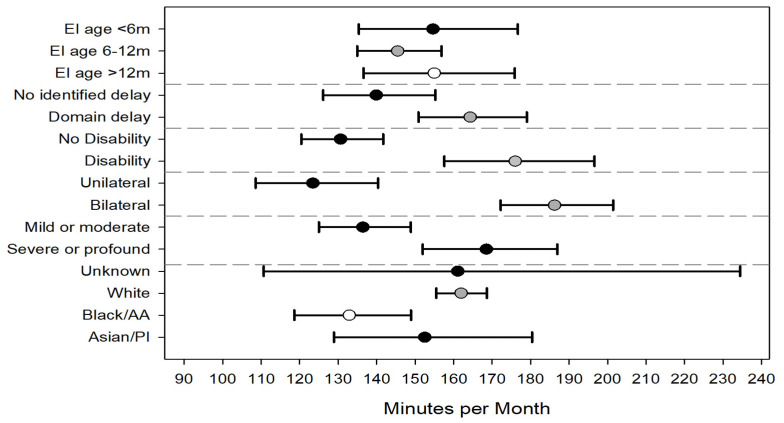
Adjusted average intensity (minutes per month) of all common early intervention service types for all 3 years combined. Error bars represent 95% confidence intervals. Error bar for unknown race extends to 234 minutes. Abbreviations: EI = early intervention, m = months, AA = African American, and PI = Pacific Islander.

Regression models were constructed to understand independent factors associated with EI intensity in the 1st year of EI and across all 3 years (see Table A2 for model parameter estimates). In the first year of EI, children were, on average, assigned more intensive therapy if they had bilateral hearing loss compared to unilateral (153 vs. 116 min/month), severe or profound hearing loss compared to mild or moderate (148 vs. 120), and diagnosed with a developmental disability vs. no disability (143 vs 124). Enrolling into EI by 6 months of age was associated with significantly lower intensity than enrolling into EI after 12 months of age (120 vs. 149) (Figure 4). Though the average intensity increased over time, the model findings from the first EI year were consistent with the model that included data from all 3 years. Age of EI enrollment was no longer statistically significant in the model including all years. (Figure 5).

Variables, such as the presence of a hearing loss risk indicator at birth and premature birth, were not statistically significant in the models. Infants whose race was reported on the birth certificate as black/African American had significantly lower EI intensity compared to infants whose race was reported as white. No statistically significant differences were noted across the other race categories. Hispanic ethnicity was not significant in any of the models. There was great variability across the 9 regions of EI delivery, with intensity ranging from 139 (95% CI 123–158) minutes per month to 168 (95% CI 151–187) minutes per month. There did not appear to be a relationship between EI regions serving predominantly rural areas and service intensity. Two regions serving rural counties had intensities of 139 and 167 minutes per month.

### 3.4. Service Intensity–DHH Service

Of the 1262 infants and toddlers who received EI, 833 (66%) received the DHH service type at least once during EI. The median (interquartile range [IQR]) for DHH service intensity at the first IFSP (minutes per month) was 60.0 [45.0–60.0]. Median intensity for DHH service type did not increase with the first year of EI (59.1 [45.0–60.0] minutes per month) and was similar across all years of EI service (53.0 [42.1–60.0] minutes per month). In the unadjusted analysis, children with bilateral hearing loss and children who enrolled in EI after 12 months of age had significantly higher intensity of specialized DHH services (Table 4). This finding was consistent for the first year of EI service as well as across all 3 years. 

**Figure 6:**
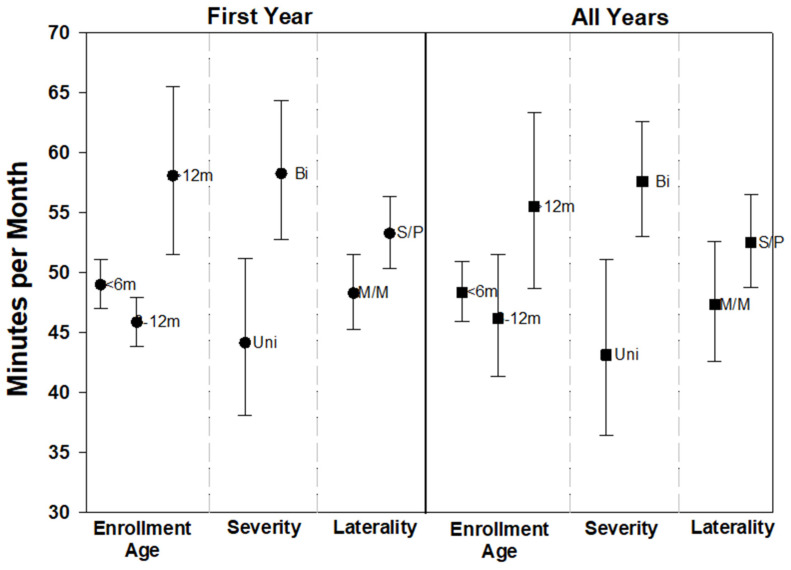
Adjusted average intensity (minutes per month) for specialized DHH services only for the first enrollment year and all years combined. Error bars represent 95% confidence intervals. Abbreviations: m = months, Uni = unilateral, Bi = bilateral, M/M = mild or moderate, and S/P = severe or profound.

Results of the regression models for the first year and all 3 years were consistent with each other (See Table A2). Both models found bilateral hearing loss (vs. unilateral) and severe or profound levels of hearing loss (vs. mild or moderate) significantly associated with higher intensity for DHH service type. Children who entered EI after 12 months of age had significantly higher DHH service intensity compared to the enrollment age categories of <6 months and 6–12 months. (Figure 6).

## 4. Discussion

The present study quantified the intensity of EI services, defined as minutes per month, that were assigned to infants and toddlers who were identified as deaf or hard of hearing from a large populous state. Focusing on the most common services listed in the IFSP, children received on average over 2 h (138 min) per month of EI service across all years and less than an hour (53 min) per month of Specialized DHH services. We found that greater service intensity was associated with having bilateral hearing loss, severe or profound hearing loss, a diagnosis of a developmental disability, and older age of EI enrollment.

This work extends our knowledge about EI for children who are DHH beyond the age of enrollment to include an understanding of EI service intensity. Our study findings are consistent with previous studies on EI intensity; DHH children received a little over 2 h per month with substantial variability in intensity; 10% of children received at least 2 h per week. Previous literature has stated that children enrolled in EI receive a range of service intensity from approximately 2 h per week [18] to 2–3 h per month [19,20,21]. Wiggen et al. [12] reported that children who were DHH received on average 3–4 intervention sessions per month ranging from 30–90 min, with the majority of sessions lasting 60 min each. Intervention sessions were not necessarily limited to Part C EI and included sessions that occurred within intervention centers. For children who are DHH, early interventions are designed to minimize or prevent delays and promote language development. Thus, our EI cohort differed from other studies in that EI eligibility was based on identified hearing loss, irrespective of any developmental disabilities or delays that may have been present. 

### 4.1. Factors Associated with Intensity of Services

Many factors may influence the intensity of EI services, including state variability in programming, sample or population, and individual child or family characteristics. Contextual factors, such as family income, SES, and geographic location have all been associated with EI service (either intensity or accessibility) [19,22,23]. Our study results suggest specific factors may influence the intensity of EI services for children who are DHH. The presence of a diagnosed disability (26% of cohort) and/or an identified delay (33% of cohort) across any of the five domains was both associated with increased EI service intensity, which is consistent with previous literature findings that developmental needs are a key driver of service intensity [20,24]. Because children with additional developmental needs likely require additional intervention service lines, the number of different services and amount of time spent in EI services could be higher for this subgroup. In fact, the majority of studies on EI intensity have occurred in populations of children who have developmental concerns or known diagnoses impacting development. Our focus on children who are DHH makes this research unique compared to others, as our target population was eligible for EI because of an identified hearing loss and not necessarily due to other developmental delays or risks. This may influence service delivery, as EI programming for infants and toddlers who are DHH is intended to support the proactive development of skills rather than ameliorate existing delays. EI services for children who are DHH are readily available across the state and a regionalized approach is used to provide specialized expertise in the early development of infants and children who are DHH.

Several studies have linked contextual factors to EI service intensity, though findings have not always been consistent. A number of studies have linked income levels with service intensity, with higher income associated with higher intensity, more hours of service, or increased number of services [19,20,22,23]. Health insurance has also been associated with EI intensity, though findings have not been consistent. Khetani et al. [19] found that children receiving public insurance had lower intensity measures while Hallam et al. [22] found that children who were Medicaid eligible had higher intensity measures. Although our study did not have family income level, we were able to evaluate the health insurance at the time of the child’s birth and found no relationship with EI service intensity.

In our cohort, older age at EI enrollment was significantly associated with higher overall service intensity in year 1, but not associated across all years. We also found that EI enrollment after 12 months of age was also associated with higher DHH service intensity compared to enrollment before age 12 months. These findings are consistent with previous studies that have demonstrated that older enrollment or entry ages are associated with higher levels of services [20,21,23]. We believe our findings regarding enrollment age are partly due to the eligibility of children who are DHH for EI (irrespective of any other diagnoses). Children enrolled in infancy may not have many other developmental needs and therefore the purpose of EI may be as a prevention measure to ensure appropriate language development. However, children who enroll at later ages may be entering EI with more delayed developmental outcomes and therefore require more intense services (more time in service and more service types) to try and “catch up” prior to EI exit that is required at 36 months of age. Consistent with a previous work [12], we found that children with either bilateral or more significant degrees of hearing loss had levels of higher service intensity. Children with more severe levels of hearing loss may require additional services, given the potential greater impact their hearing loss may have on language development, compared to those with milder levels. It is important that young children with unilateral hearing loss of any degree and mild bilateral hearing loss are still identified early and offered appropriate EI services as soon as possible [25]. These services should include the same services offered to all children who are DHH, even though the intensity of services may be less [26].

At the time EI was delivered to children who were DHH, EI was provided in Ohio across nine regions with significant variability in service intensity, ranging from 139 to 168 min per month. We did not find a relationship with regions that were made up of rural counties (with intensity measures in the extreme) nor did we find this with regions that included our most populous counties (the three regions included with EI intensity measured between 141 and 156 min per month). It has been demonstrated that individuals living in low-income neighborhoods experience inequities regarding access to care and receipt of timely EI services [27]. When focusing on rural vs. urban areas, young children residing in rural areas may receive fewer services compared to urban areas, despite poverty levels [24]. In our study, we did not see consistent intensity across predominantly rural regions. It is possible that in some regions, access to other services for needs (e.g., private therapies) may be limited and the reliance on Part C EI services becomes more necessary for families in these regions. It is also possible that access to trained staff may be challenging in rural counties, making it difficult to provide as much time per month of certain services as others. In Ohio, specialized DHH services were centralized by region, making access to this service possibly more uniform within a region, but introducing variability across regions. 

We found that infants whose race was identified as either black or African American on the birth certificate, compared to infants identified as white, had significantly lower intensity levels (approximately 20% lower) for all common EI service types. We did not find this association when focusing on the specialized DHH services. EI disparities have been demonstrated to exist in previous studies focused on EI access [28,29,30]. Families who are minorities may face unique barriers regarding access to EI services [28,31,32]. Barriers to accessing health services are multifaceted and likely include structural or systemic barriers that are long standing. More targeted research is needed to better understand these barriers so that we can ensure equitable access to EI services.

### 4.2. Limitations

Because this was a retrospective study that used public health data, there are several limitations. We had available only services that were authorized on the IFSP; we did not have whether the services were actually accessed by families. Thus, it is possible we overestimated exposure with the assumption that families received precisely what was listed in the IFSP. It is also possible that children received interventions outside of the Part C program, which were not captured. Audiology services within the IFSP did not include services that were received outside of Part C. Therefore, the actual receipt of audiology services is very likely highly under-represented in this study. We also were unable to capture the quality of the services nor the level of parental engagement, as this information is not reported within the IFSP. Although we had data on whether a child had a disability diagnosed by a medical professional, we had no other details regarding the disability.

Within this study, the race and ethnicity of a child came from the birth certificate, which included a specific list of possible racial categories; thus there were limitations with definition and classification. Additionally, a large number of infants were reported with the category of “other” with no attached explanation in our dataset. Because “other” was equivalent to “unknown”, we collapsed these categories, further making interpretation of this category difficult. At best, this cursory and crude evaluation of race in this study provided us with some possible ideas regarding how to conceptualize potential structural or societal barriers to EI services as we move forward. We did not see significant associations between intensity and outcomes by ethnicity, though the small numbers of infants classified as ‘Hispanic’ made understanding this relationship problematic and requires a more in-depth investigation around EI across different cultures and ethnicities. It is important to also keep in mind that our findings are specific to Ohio and although they may be generalizable to some degree, each state has its own rules regarding how intervention services are allocated across different systems. This study represents one state system. Services in other regions may be allocated differently across different systems in different states, and thus our results may not be fully generalizable.

### 4.3. Future Directions

Although prior studies have shown an association between higher EI service intensity and positive changes or improvements in outcomes, optimal intensity is relatively unknown. Higher dosage or intensity levels of Part C EI have been linked to higher adaptive skills (communication, socialization, and daily living skills) [23] and positive changes in functional gains when EI services ended [21]. For infants and toddlers who are DHH, EI plays a prominent role by providing services that support language and other skills important for growth and development. Research has historically focused on outcomes associated with age of EI enrollment; earlier exposure to EI is associated with better outcomes in both the short-term (language and vocabulary) [8,9,10,11] and longer-term (early academic outcomes) [16,33]. However, few reported studies have evaluated intensity measures in this population. Geers et al. found that more intervention hours before 36 months of age were associated with higher language in preschool, though intervention in that study was provided through a deaf education center [13]. Chu et al., reported that lower doses of EI were associated with better expressive language in children with cochlear implants, though the results were confounded by major group differences [14]. By understanding measures of service intensity within Part C, we can begin evaluating the association with outcomes for young children who are DHH. Broadening our understanding of the components and structure of Part C services that are effective can help state EI programs focus necessary resources for robust child and family supports. Additionally, future research focusing on the intensity and quality of services as well as child developmental needs and family factors will be helpful to refine service provision and ensure all children who are DHH have the opportunity to thrive. 

## Figures and Tables

**Table 1:** Characteristics of infants/toddlers who were deaf or hard of hearing served in early intervention.

Characteristic	Total *N* = 1262
Gender–Female	578 (45.8%)
Race	
Black/African American	155 (12.3%)
White	967 (76.6%)
Asian/Pacific Islander	30 (2.4%)
Other/Unknown	110 (8.7%)
Hispanic ethnicity Unknown ethnicity	56 (4.4%) 101 (8.0%)
Premature birth	270 (21.4%)
Private insurance Unknown	600 (47.5%) 115 (9.1%)
Higher maternal education ^1^ Unknown	712 (56.4%) 132 (10.5%)
Higher paternal education ^1^ Unknown	568 (45%) 297 (23.5%)
Presence of a disability diagnosis	323 (25.6%)
Bilateral hearing loss	954 (75.6%)
Severe or profound hearing loss	445 (35.3%)
Has at least one risk indicator for hearing loss	507 (40.2%)
Age confirmed hearing loss	3.9 (1.9–9.0)
Age enrollment into EI	5.3 (3.2–9.3)
Age at EI enrollment	
<6 months	713 (56.5%)
6 to <12 months	310 (24.6%)
≥12 months	239 (18.9%)

*N* with percentages in parentheses or medians with interquartile range in parentheses. ^1^ Some college education at time of child’s birth.

**Table 2:** Child characteristics and service use.

Characteristic	*N* = 1262
Child has identified skill delays ^1^	
Communication	354 (28.1%)
Cognitive	165 (13.1%)
Motor	214 (17%)
Social	92 (7.3%)
Adaptive	156 (12.4%)
In any domain	417 (33.0%)
Identified skill delay in any domain in first year	356 (28.2%)
Has a documented diagnosed disability (on physician form)	323 (25.6%)
Number of common EI services received in year ^1^	
1	60 (4.8%)
2	261 (20.7%)
3	355 (28.1%)
4 or more	586 (46.4%)
Number of common ^2^ EI services received over entire EI	
1	45 (3.6%)
2	180 (14.3%)
3	277 (22%)
4 or more	760 (60.2%)
Cumulative hours of total core service over entire EI, Median (interquartile range) Mean (SD)	56.8 (28.4–111.7) 99.4 (157.0)

^1^ Identified at any time while in early intervention (EI). ^2^ Services included service coordination, specialized DHH services, special instruction, speech-language therapy, family training and counseling, physical therapy, occupational therapy, audiologic services, and parent education.

**Table 3:** EI intensity (minutes per month) for all selected services listed in the first IFSP, in the year, and across all years. Data reported as medians with interquartile range in parentheses.

	At 1st IFSP	*p* ^1^	In 1st Year	*p* ^1^	Across All Years	*p* ^1^
For all children	73.3 (48.0–126.7)		121.2 (75.6–204.6)		138.1 (82.3–262.1)	
Age of EI enrollment		0.041		0.055		0.40
<6 months (*n* = 713)	70.9 (46.2–120.0)		118.4 (75.3–187.0)		142.5 (85.1–268.4)	
6 to <12 months (*n* = 310)	80.3 (50.3–140.3)		130.1 (79.6–225.9)		136.0 (81.1–258.7)	
≥12 months (*n* = 239)	70.9 (45.0–135.4)		128.7 (70.9–242.8)		134.2 (70.9–256.0)	
Race		<0.0001		0.13		0.20
Black/African American	60.0 (30.4–98.8)		111.2 (65.9–168.5)		132.7 (69.5–214.7)	
White	80.3 (50.7–129.2)		123.3 (79.5–207.6)		141.0 (85.3–273.8)	
Asian/Pacific Islander	68.0 (45.6–130.9)		109.2 (70.1–180.0)		127.7 (76.7–262.1)	
Other/Unknown	74.9 (50.7–146.9)		122.9 (70.9–229.0)		138.9 (78.9–263.9)	
Ethnicity		0.29		0.13		0.22
Hispanic	65.4 (27.6–120.0)		95.1 (59.3–188.3)		132.7 (72.0–240.2)	
Non-Hispanic	72.6 (48.1–122.4)		122.3 (77.1–204.0)		140.3 (84.3–265.5)	
Gestation		0.42		0.007		0.002
Premature	70.1 (38.8–130.1)		138.9 (84.9–230.9)		156.4 (100.2–283.8)	
Term	73.5 (49.7–120.9)		119.5 (73.5–193.6)		132.8 (79.6–255.6)	
Insurance status (at birth)		0.27		0.31		0.22
Has private insurance	71.8 (48.1–120.0)		119.5 (75.3–192.8)		132.2 (81.0–263.4)	
Has public insurance	70.9 (46.4–131.7)		123.4 (77.1–210.0)		142.3 (86.2–270.6)	
Hearing loss risk indicator		0.003		0.036		0.0001
Has risk indicator	67.6 (35.5–124.1)		126.7 (79.7–233.1)		157.8 (90.0–303.1)	
No risk indicator	80.3 (50.7–130.0)		120.0 (71.5–189.3)		128.6 (77.7–240.1)	
Maternal education level		0.04		0.19		0.19
Some college	70.9 (45.6–120.0)		118.6 (74.3–205.5)		132.9 (79.4–261.1)	
Less than college	81.1 (50.4–131.7)		127.0 (80.6–204.3)		145.0 (90.4–270.8)	
Paternal education level		0.0002		0.003		0.004
Some college	70.1 (45.6–120.0)		116.0 (71.3–188.5)		128.2 (77.1–251.4)	
Less than college	84.4 (55.7–140.1)		130.9 (86.2–223.7)		154.4 (92.9–288.3)	
Degree of hearing loss		0.24		0.0004		0.0002
Severe or profound	76.4 (49.4–135)		132.7 (87.1–230.2)		160.0 (96.4–289.1)	
Mild or moderate	70.9 (46.8–120)		118.1 (70.9–189.0)		130.1 (77.7–246.7)	
Laterality		0.002		<0.0001		<0.0001
Bilateral	80.3 (50.3–130.1)		128.8 (81.7–212.4)		153.0 (91.1–293.0)	
Unilateral	60.8 (43.9–115.7)		98.9 (60.8–163.0)		103.5 (64.6–182.8)	
Presence of disability ^2^		0.18		0.02		<0.0001
Yes	65.9 (40.9–125.1)		132.7 (80.6–23.1)		176.4 (99.0–248.1)	
No	76.4 (50.3–126.7)		120.0 (72.0–195.5)		130.1 (79.7–231.6)	
Identified delay in any domain ^3^		0.14		0.02		0.016
Yes	70.1 (37.8–131.7)		126.4 (76.1–204.6)		145.0 (89.7–293.0)	
No	75.2 (50.7–121.6)		120.0 (75.3–186.6)		135.0 (80.8–248.6)	

^1^*p*-value derived from Wilcoxon Sum Rank test. ^2^ Presence of a disability that places child at high risk for developmental delays. ^3^ Domain delays identified at any time during EI.

**Table 4:** EI intensity (minutes per month) for DHH services listed in the first IFSP, in the year, and across all years. Data reported as medians with interquartile range in parentheses.

	At 1st IFSP *N* = 486	*p* ^1^	In 1st Year *N* = 756	*p* ^1^	Across All Years *N* = 833	*p* ^1^
For all children	60.0 (45.0–60.0)		59.1 (45.0–60.0)		53.0 (42.1–60.0)	
Age of EI enrollment		0.07		0.026		0.011
<6 months (*n* = 713)	60.0 (45.0–60.0)		59.6 (45.0–60.0)		52.9 (40.7–60.0)	
6 to <12 months (*n* = 310)	50.7 (38.0–60.0)		54.5 (41.9–60.0)		50.8 (41.2–60.0)	
≥12 months (*n* = 239)	60.0 (40.5–60.8)		60.0 (45.7–76.0)		60.0 (45.6–76.3)	
Race		0.73		0.99		0.87
African American/Black	55.3 (30.4–60.0)		60.0 (45.0–60.0)		54.2 (45.0–60.0)	
White	54.2 (45.0–60.0)		59.1 (45.0–60.0)		53.3 (41.6–60.0)	
Asian/Pacific Islander	50.7 (38.0–60.0)		50.7 (43.2–60.0)		50.7 (37.1–60.0)	
Unknown	50.7 (38.0–60.0)		58.1 (42.2–60.0)		52.6 (42.9–60.0)	
Ethnicity		0.09		0.07		0.32
Hispanic	50.7 (30.4–50.7)		50.7 (34.3–52.1)		50.7 (42.6–54.8)	
Non-Hispanic	60.0 (45.0–60.0)		59.4 (45.0–60.0)		53.0 (42.1–60.0)	
Gestation		0.12		0.72		0.86
Premature	60.0 (45.0–60.0)		60.0 (43.3–60.0)		54.3 (41.5–60.0)	
Term	50.7 (45.0–60.0)		55.6 (45.0–60.0)		52.3 (42.2–60.0)	
Insurance status (at birth)		0.91		0.62		0.54
Has private insurance	50.7 (45.0–60.0)		55.2 (44.6–60.0)		52.3 (41.0–60.0)	
Has public insurance	60.0 (38.0–60.0)		60.0 (45.0–60.0)		54.1 (43.2–60.0)	
Hearing loss risk indicator		0.44		0.47		0.40
Has risk indicator	60.0 (38.0–60.0)		60.0 (45.0–60.0)		55.9 (43.5–60.0)	
No risk indicator	50.7 (45.0–60.0)		55.0 (44.7–60.0)		51.7 (41.5–60.0)	
Maternal education level		0.58		0.65		0.41
Some college	60.0 (45.0–60.0)		58.6 (45.0–60.0)		52.6 (40.5–60.0)	
Less than college	50.7 (38.0–60.0)		52.3 (43.4–60.0)		52.1 (43.2–60.0)	
Paternal education level		0.81		0.17		0.19
Some college	50.7 (45.0–60.0)		55.2 (43.2–60.0)		51.1 (39.9–60.0)	
Less than college	60.0 (45.0–60.0)		60.0 (45.0–60.0)		55.3 (43.9–60.0)	
Degree of hearing loss		0.99		0.59		0.13
Severe or profound	50.7 (45.0–60.0)		55.2 (45.0–60.0)		53.3 (44.6–60.0)	
Mild or moderate	60.0 (38.0–60.0)		60.0 (44.7–60.0)		53.0 (40.5–60.0)	
Laterality of hearing loss		<0.0001		<0.0001		<0.0001
Bilateral	60.0 (45.0–60.0)		60.0 (45.6–60.0)		58.3 (45.3–60.0)	
Unilateral	50.7 (14.0–60.0)		45.6 (27.1–60.0)		45.6 (26.4–60.0)	
Has diagnosed disability ^2^		0.37		0.47		0.73
Yes	60.0 (45.0–60.0)		55.5 (44.0–60.0)		52.6 (40.6–60.0)	
No	50.7 (38.0–60.0)		59.4 (45.0–60.0)		53.2 (42.7–60.0)	
Identified delay in any domain ^3^		0.46		0.43		0.40
Yes	50.7 (45.0–60.0)		50.7 (45.0–60.0)		50.7 (43.2–60.0)	
No	60.0 (45.0–60.0)		60.0 (44.6–60.0)		56.1 (40.7–60.0)	

^1^*p*-value derived from Wilcoxon Sum Rank test. ^2^ Presence of a disability that places child at high risk for developmental delays. ^3^ Domain delays identified at any time during EI.

## Data Availability

Data used for this research are not publicly available due to the legal issues regarding data collected as part of a statewide program specific to a subgroup of children who are deaf or hard of hearing served in early intervention.

## Appendix A

**Table A1:** Components of early intervention specifically for deaf or hard of hearing children.

Components
Home-based family support
Unbiased parent education on communication choices
Assistance with follow-up audiological appointments, and connections to community resources
Guidance in communication and language development
Opportunities to interact with the D/deaf community
Parent-to-parent support
Planning for transition to preschool

## Appendix B

**Table A2:** Results from mixed models of intensity for all common early intervention (EI) services and specialized DHH services.

	All Common EI Services				DHH Services			
Variable	1st Year		All 3 Years		1st Year		All 3 Years	
	Parameter estimates (SE)	*p*-value	Parameter estimates (SE)	*p*-value	Parameter estimates (SE)	*p*-value	Parameter estimates (SE)	*p*-value
Age of EI enrollment <6 months 6–12 months >12 months	−0.21 (0.07) −0.11 (0.06) Ref	0.001 0.046	−0.002 (0.07) −0.06 (0.06) Ref	0.98 0.30	−0.17 (0.06) −0.24 (0.07) Ref	0.002 0.001	−0.14 (0.06) −0.18 (0.05) Ref	0.018 0.0006
Race Asian/PI Black/African American Other/Unknown White	−0.02 (0.09) −0.20 (0.08) −0.14 (0.19) Ref	0.78 0.01 0.47	−0.06 (0.10) −0.20 (0.06) −0.01 (0.19) Ref	0.53 0.0005 0.98	0.06 (0.08) −0.002 (0.04) −0.11 (0.03) Ref	0.48 0.96 0.0008	0.009 (0.07) −0.03 (0.04) −0.05 (0.07) Ref	0.90 0.46 0.42
Delay identified in any domain	0.13 (0.03)	0.0001	0.16 (0.04)	0.005	---	---	---	---
Presence of disability diagnosis	0.18 (0.06)	0.003	0.30 (0.05)	<0.0001	---	---	---	---
Laterality Bilateral Unilateral	0.27 (0.05) Ref	<0.0001	0.41 (0.06) Ref	<0.0001	0.28 (0.12) Ref	0.02	0.29 (0.11) Ref	0.007
Degree of hearing loss Severe or profound Mild or moderate	0.21 (0.04) Ref	<0.0001	0.21 (0.04) Ref	<0.0001	0.10 (0.04) Ref	0.02	0.10 (0.04) Ref	0.012

Abbreviations: EI = early intervention, DHH = deaf or hard of hearing, SE = standard error, PI = Pacific Islander, and Ref = reference.
